# Supplementary material for: Trajectories of atherosclerotic cardiovascular disease risk scores as a predictor for incident chronic kidney disease
Source: BMC Nephrol. 2024 Apr 22;25:141. doi: 10.1186/s12882-024-03583-1 (PMC11036697; doi:10.1186/s12882-024-03583-1)
Supplement: Supplementary file 1 — Supplementary Material 1. [file 12882_2024_3583_MOESM1_ESM.docx]

**Supplementary Table 1**. Estimation process for the trajectory groups of determined by the 6-year ASCVD risk score measurements.

| Number of groups | BIC | Groups | | | | |
| --- | --- | --- | --- | --- | --- | --- |
|  |  | 1 | 2 | 3 | 4 | 5 |
| 2 | 99071.14 | 83.82 | 16.18 |  |  |  |
| 3 | 99096.71 | 94.46 | 0 | 5.54 |  |  |
| 4 | 99122.28 | 5.78 | 1.53 | 0 | 92.69 |  |
| 5 | 99147.85 | 7.99 | 0 | 0 | 92.01 | 0 |

Abbreviations: ASCVD, atherosclerotic cardiovascular disease; BIC, Bayesian information criterion.

**Supplementary Table 2.** Sensitivity analysis for the risk of incident CKD among individuals who consistently participated in the study during the exposure period.

|  | Stable ASCVD risk | Increasing ASCVD risk | | |
| --- | --- | --- | --- | --- |
| Total cases, n | 3844 | 790 | | |
| Incident CKD cases, n | 285 | 126 | | |
| Follow-up time, person-year | 36957.0 | 7302.7 | | |
| Incident rate per 1000 person-year | 7.71 | 17.25 | | |
|  |  | HR | 95% CI | *p* |
| Unadjusted | 1 (reference) | 2.25 | 1.83–2.78 | < 0.001 |
| Model 1 | 1 (reference) | 1.40 | 1.07–1.85 | 0.016 |
| Model 2 | 1 (reference) | 1.38 | 1.04–1.82 | 0.027 |
| Model 3 | 1 (reference) | 1.36 | 1.03–1.81 | 0.033 |

Model 1: Adjusted for sex, age groups, BMI, total energy intake, smoking status, alcohol drinking status, and physical activity.

Model 2: Adjusted for variables used in Model 1 plus MBP, FPG, serum total cholesterol, and CRP levels.

Model 3: Adjusted for variables used in Model 2 plus baseline ASCVD risk and baseline eGFR.

Abbreviations: ASCVD, atherosclerotic cardiovascular disease; CKD, chronic kidney disease; BMI, body mass index; MBP, mean blood pressure; FPG, fasting plasma glucose; CRP, C-reactive protein; eGFR, estimated glomerular filtration rate; HR, hazard ratio; CI, confidence interval.

**Supplementary Table 3.** Sensitivity analysis for the prevalence of proteinuria among individuals who consistently participated in the study during the exposure period.

|  | ASCVD risk trajectory groups | | |
| --- | --- | --- | --- |
| Prevalence of proteinuria (%) | Stable | Increasing | *p* |
| At 8 years | 1.87 | 3.87 | 0.002 |
| At 10 years | 1.70 | 3.41 | 0.005 |
| At 12 years | 3.41 | 5.68 | 0.006 |
| At 14 years | 4.11 | 8.40 | <0.001 |
| At 16 years | 6.50 | 9.96 | 0.001 |

Abbreviations: ASCVD, atherosclerotic cardiovascular disease.
